# Proteome Sampling by the HLA Class I Antigen Processing Pathway

**DOI:** 10.1371/journal.pcbi.1002517

**Published:** 2012-05-17

**Authors:** Ilka Hoof, Debbie van Baarle, William H. Hildebrand, Can Keşmir

**Affiliations:** 1 Theoretical Biology and Bioinformatics, Utrecht University, Utrecht, The Netherlands; 2 Department of Immunology, University Medical Center Utrecht, Utrecht, The Netherlands; 3 Department of Microbiology and Immunology, University of Oklahoma Health Sciences Center, Oklahoma City, Oklahoma, United States of America; La Jolla Institute for Allergy and Immunology, United States of America

## Abstract

The peptide repertoire that is presented by the set of HLA class I molecules of an individual is formed by the different players of the antigen processing pathway and the stringent binding environment of the HLA class I molecules. Peptide elution studies have shown that only a subset of the human proteome is sampled by the antigen processing machinery and represented on the cell surface. In our study, we quantified the role of each factor relevant in shaping the HLA class I peptide repertoire by combining peptide elution data, *in silico* predictions of antigen processing and presentation, and data on gene expression and protein abundance. Our results indicate that gene expression level, protein abundance, and rate of potential binding peptides per protein have a clear impact on sampling probability. Furthermore, once a protein is available for the antigen processing machinery in sufficient amounts, C-terminal processing efficiency and binding affinity to the HLA class I molecule determine the identity of the presented peptides. Having studied the impact of each of these factors separately, we subsequently combined all factors in a logistic regression model in order to quantify their relative impact. This model demonstrated the superiority of protein abundance over gene expression level in predicting sampling probability. Being able to discriminate between sampled and non-sampled proteins to a significant degree, our approach can potentially be used to predict the sampling probability of self proteins and of pathogen-derived proteins, which is of importance for the identification of autoimmune antigens and vaccination targets.

## Author Summary

HLA class I molecules are expressed on the cell surface of almost all cells of the human body in complex with short fragments (peptides) of cytosolic proteins, thereby providing a snapshot of the intracellular state of a cell to circulating CD8+ T cells. Several processes are involved in shaping the peptide ligand repertoire of an HLA class I molecule, which generally represents only a small fraction of the proteins available in the cytosol. In our work we addressed protein sampling by HLA class I molecules to answer two questions: 1) Which proteins are sampled by the antigen processing pathway and why, and 2) which peptides of a given protein are picked to represent the source protein on the cell surface? To this end we quantified the contribution of each process involved in peptide processing and presentation individually and combined them into a logistic regression model. This simple model enabled us to predict the sampling probability of self proteins and may aid in the identification of autoimmune antigens.

## Introduction

Major histocompatibility complex (MHC) class I molecules play a crucial role in the adaptive immune response of higher vertebrates. These molecules, in humans referred to as human leukocyte antigen (HLA) class I molecules, bind peptides derived from endogenous proteins of host or, in the case of infected cells, of pathogen origin and present them to circulating CD8+ T lymphocytes and natural killer (NK) cells. The presentation of self peptides by an individual's HLA class I molecules has an impact on positive and negative selection of CD8+ T lymphocytes in the thymus [1], [2], maintenance of naive T cells in the periphery [3], [4], and inhibition of NK cells through recognition of self peptides in the context of HLA class I molecules by killer cell immunoglobulin-like receptors (KIR) [5].

Generally, HLA class I ligands are derived from intracellular proteins, which are degraded by the proteasome into peptide fragments. These peptides are then translocated by the transporter associated with antigen processing (TAP) into the lumen of the endoplasmic reticulum (ER), where they may be loaded onto an HLA molecule if the peptide sequence fits the HLA molecule's binding preference. The C-terminus of an HLA ligand is assumed to be mainly determined by the proteasome (even though recently a carboxypeptidase has been found to contribute to C-terminal editing [6]), whereas the N-terminus may be trimmed by cytosolic and endoplasmic aminopeptidases after proteasomal cleavage [7]. Finally, the HLA-peptide complexes are transported to the cell surface for presentation to CD8+ T cells and NK cells.

Several studies analyzed peptide data sets obtained by peptide elution from specific cell lines and peptide sequencing by mass spectrometry to characterize the HLA peptide repertoire [8], [9], [10], [11]. Most of these studies focused on characterizing the function and subcellular localization of source proteins and suggested that HLA class I presented peptides are sampled from functionally and compartmentally diverse proteins, with a functional bias towards RNA-binding proteins [8]. In human cells, a weak correlation has been found between the abundance of HLA class I ligands presented and the corresponding mRNA levels [10], [11], whereas peptides eluted from murine thymocytes were preferentially derived from highly abundant mRNAs [12].

Here, we take a different angle to the question of what fraction of the human proteome is represented on the cell surface. We studied two large HLA ligand data sets obtained by peptide elution [13], [14] with the aim to quantify the role of several factors shaping the peptide repertoire of HLA class I molecules. We show that the gene expression level, protein abundance, rate of potential binding peptides in a protein and the processing quality of these peptides all contribute to which proteins are sampled and which peptides are chosen to be presented on the cell surface. Having studied the impact of each of these factors separately, we subsequently combined all factors in a logistic regression model in order to quantify their relative impact. This model can potentially be used to predict the sampling probability of self proteins and of pathogen-derived proteins.

## Results

### Elution data sets and procedure to determine HLA binders

We studied two different peptide elution data sets. One large set, which we will call the *Johnson* data, comprises 4717 human peptides and 105 vaccinia peptides eluted from vaccinia infected cells [13]. A second set of eluted peptides, the *Ben Dror* data, comprises 569 human peptides eluted from soluble HLA-B*27:05 (see Materials and Methods for details) [14]. By mapping each eluted peptide to the human proteome, we were able to uniquely identify the source protein for 90% (4243 of 4717) of the *Johnson* data and 81.9% (466 of 569) of the *Ben Dror* data. Peptides that mapped to several human proteins (about 9%) or for which no source protein could be identified (only 0.4–1.1%) were excluded from further analysis.

**Figure 1:**
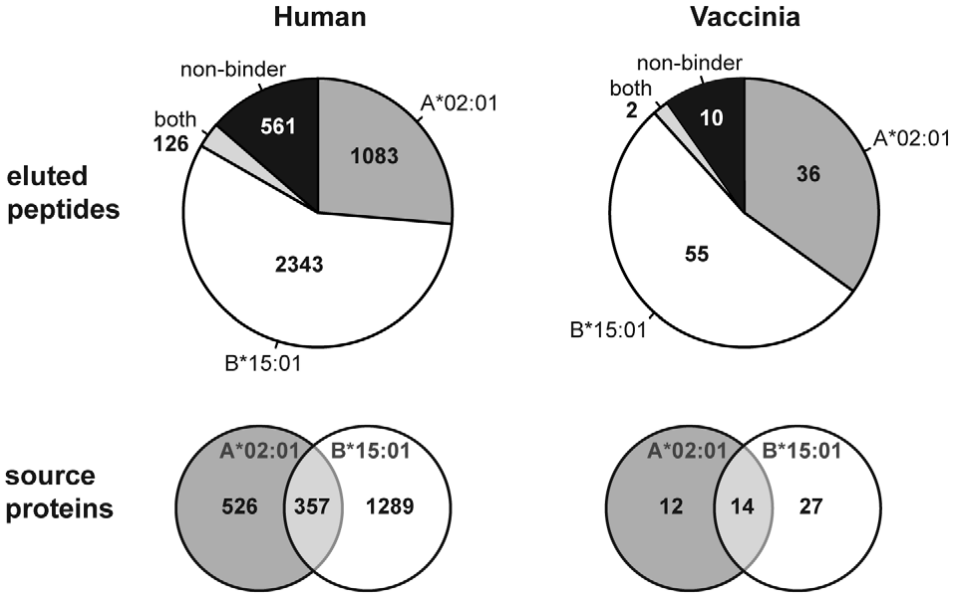
Composition of the Johnson data. The pie charts depict the fractions of eluted peptides that were predicted to bind to HLA-A*02:01, B*15:01, both, or neither of the two alleles. Predictions were only performed for peptides of 8–13 amino acids in length (*n* = 4113 for human-derived peptides, *n* = 103 for vaccinia). The Venn diagrams indicate the number of source proteins these peptides originated from.

The cell line used for the generation of the *Johnson* data was homozygous for HLA-A*02:01, B*15:01 and C*03. In order to assess which of these three HLA molecules each of the reported peptides was eluted from, we employed NetMHC 3.2 [15], [16], a tool for HLA-peptide binding prediction. This tool is applicable for peptides of 8 to 13 amino acids in length [15]. Of all eluted peptides of appropriate length and that could be mapped uniquely to a human source protein (4113 of 4243 peptides), we were able to assign 86.4% (3552 of 4113) to either being eluted from A*02:01 or B*15:01. The remaining 561 peptides could potentially have been eluted from C*03. Binding predictions, however, suggested C*03 binding only for a minor fraction of these (28% for all peptide lengths and 37% for 9mers), and therefore we decided to exclude these peptides from further analysis. Surprisingly, we identified twice as many potential B*15:01 binders as A*02:01 binders, originating from a larger number of source proteins (Fig. 1). This observation is in agreement with the estimation given by the original study [13], in which the assignment to the restricting HLA molecule was solely determined based on the C-terminal residues of the eluted peptides. Likewise, among the vaccinia-derived peptides, a larger number of peptides (1.5-fold) were eluted from B*15:01 than from A*02:01, mapping to a larger number of vaccinia proteins, even though in this case the difference was less pronounced (Fig. 1).

Of the 569 eluted peptides in the *Ben Dror* data, 466 peptides (81.9%) mapped uniquely to 396 human source proteins. Among these peptides, 420 (90.1%) were predicted to bind to HLA-B*27:05, the soluble HLA molecule expressed by the cells studied. For all three HLA alleles studied, the majority of sampled proteins were represented by only a single peptide: 86.9% (457 of 526) of the proteins sampled by A*02:01, 76.1% (981 of 1289) of the ones sampled by B*15:01, and 86.4% (342 of 396) of the proteins sampled by B*27:05 gave rise to only one eluted peptide.

In total, the two elution data sets had 160 source proteins in common. GO-term enrichment analysis (see Materials and Methods) revealed that for this set of proteins biological processes relating to the cell cycle and its regulation as well as nucleic acid metabolic processes were overrepresented.

### Eluted peptides are characterized by higher binding affinity and more efficient processing

The observation that a protein is represented on the cell surface by one or more peptides allows the assumption that the protein must have been available in sufficient amounts or must have been present at an accessible subcellular location to be available for the antigen processing machinery. What factors then determine which of the potential HLA binders of a given protein will be found on the cell surface?

**Figure 2:**
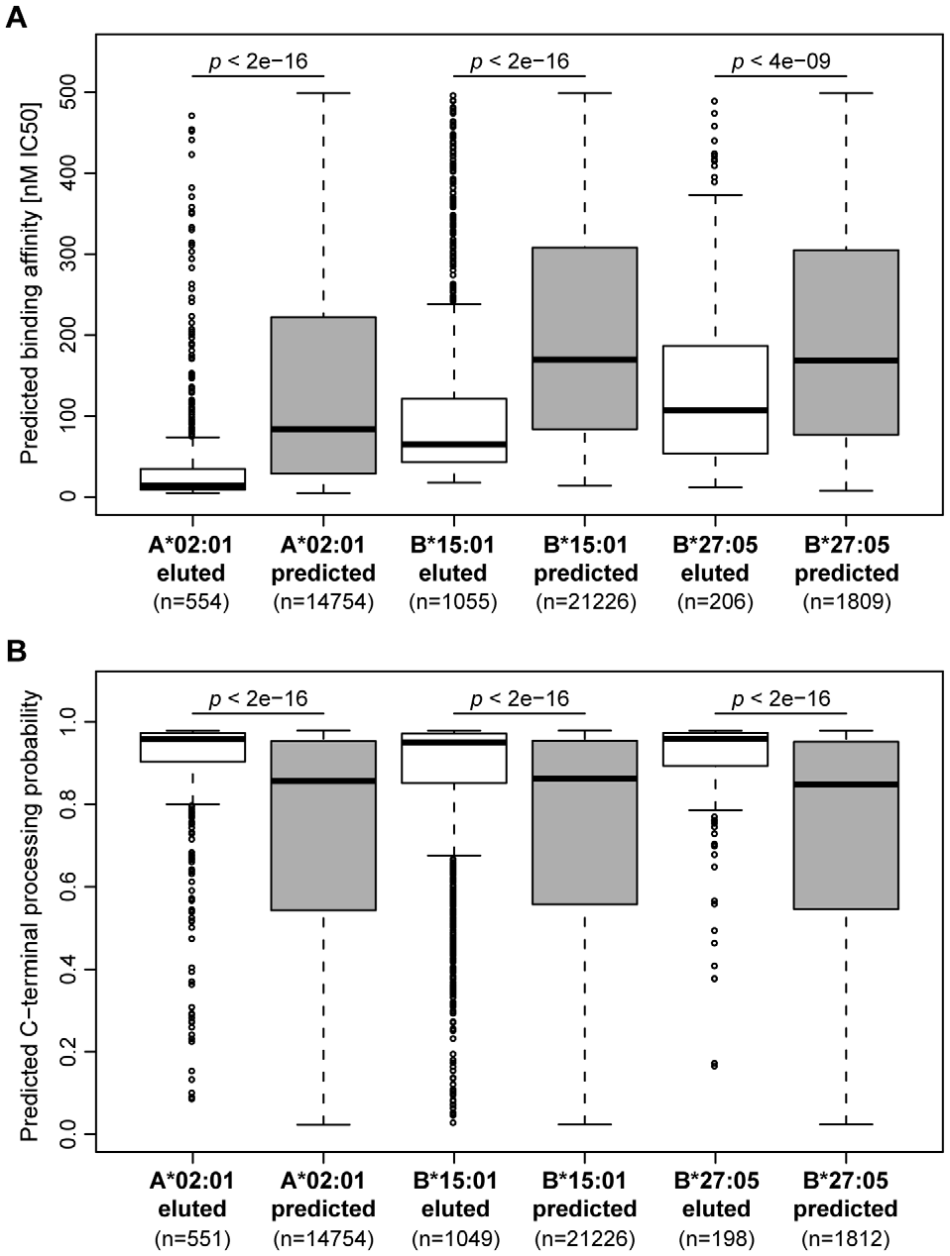
Eluted peptides show higher binding affinity to HLA and more efficient C-terminal processing. The boxplots compare eluted 9mer peptides and predicted binders from the same set of source proteins in terms of (**A**) predicted binding affinity to A*02:01, B*15:01, and B*27:05, respectively, and (**B**) predicted C-terminal processing probability. For the matter of correctness, we removed eluted peptides from the data set that had also been part of the NetChop training set (*n* = 15).

In order to characterize the obtained peptide set, we employed prediction methods for HLA binding and antigen processing (see Materials and Methods). For the identified source proteins, we predicted all potential (9mer) binders to HLA-A*02:01, B*15:01, and B*27:05 and compared the predicted binding affinity of the eluted peptides (which form a subset of all potential binders) with the predicted binders from the same source protein that were not found in the elution. To ensure an unbiased comparison, the set of eluted peptides was limited to 9mers that were predicted to bind to the respective HLA molecule. We found that eluted peptides bind their HLA molecule with a significantly higher (predicted) binding affinity than other potential binders (Fig. 2A).

However, not all predicted high-affinity binders were found in the elution. In order to investigate whether this observation may be due to inefficient processing of these peptides, we predicted the probability of C-terminal processing (using NetChop [17]) for all eluted peptides and all predicted binders that were not found in the elution data set. Based on these predictions, the set of eluted peptides is indeed more likely to arise from antigen processing as compared to the set of predicted binders (Fig. 2B). Possibly due to co-evolution between HLA class I molecules and the immunoproteasome [18], predicted binding affinity and C-terminal processing probability show a weak (but significant) correlation (Kendall's tau = −0.065, *p*-value<0.0001). Therefore, we investigated the effect of processing without the influence of HLA binding by comparing the eluted peptide set to an affinity-matched subset of predicted binders to ensure that eluted and predicted peptides show the same distribution of binding affinities (Fig. S1A). Also for this subset of predicted binders we observed a significantly lower C-terminal processing probability (Fig. S1B). NetChop was trained on the C-termini of known HLA ligands and therefore predicts the combined effect of proteasome and TAP. Investigating the impact of these two processes separately (by employing prediction methods that are part of mhc-pathway [19], [20]) suggested that the C-termini of eluted peptides are more likely to be produced by the immunoproteasome and that these peptides are more efficiently transported by TAP (Fig. S2).

For non-self peptides, we observed the same characteristics. Eluted peptides that originated from vaccinia proteins showed a significantly higher binding affinity to the respective HLA allotype than other potential binders derived from the same set of proteins (Fig. S3A). A difference in predicted C-terminal processing probability between eluted and other peptides was, however, only found for A*02:01-binding vaccinia peptides (Fig. S3B). Interestingly, we did not observe a difference in predicted HLA binding affinity between the eluted peptides that originate from human proteins and vaccinia proteins (results not shown). This is in line with an earlier study, which showed that the HLA alleles analyzed here do not show a preference for presentation of non-self over self peptides, while others, foremost HLA-A alleles, do [21], [22].

### Protein abundance impacts protein sampling rate

**Figure 3:**
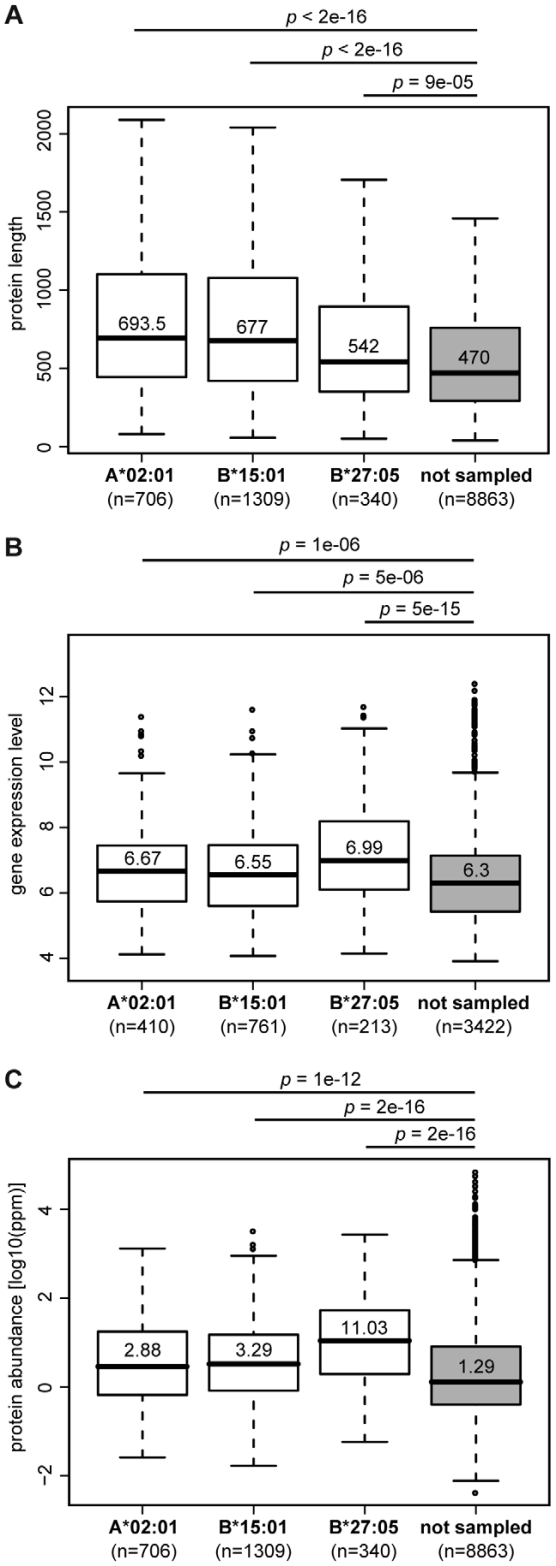
Protein length, gene expression level, and protein abundance impact protein sampling probability. The boxplots compare sampled and non-sampled human proteins in terms of (**A**) protein length, (**B**) gene expression level, and (**C**) protein abundance. The difference in protein counts between plots is due to lack of (gene expression or protein abundance) data for some of the proteins.

After having investigated what factors determine which peptides of a given protein are chosen to be presented, we turned to investigate which features of a protein impact protein sampling itself. In other words, why are some proteins sampled while others are not? Previous studies have shown that proteins giving rise to HLA ligands are foremost intracellular, distributed over various intracellular compartments with a slight bias towards the cytosol [8], [23]. Predicting the subcellular localization of each of the sampled proteins in the two data sets of our study, we found similar results: Overall, the distribution of cellular compartments for the sets of source proteins significantly differed from the distribution for the complete human proteome (Fig. S4), and specifically, extracellular proteins were significantly underrepresented in both elution data sets (*p*<1e-09, Chi-squared test), while proteins resident in the cytosol were overrepresented (*p*<2e-05). In addition, we tested several protein characteristics for their ability to discriminate source proteins from proteins that were not sampled by the antigen processing pathway. For all three HLA allotypes studied, source proteins are longer, more abundant, and the corresponding genes are more highly expressed (Fig. 3). These factors, however, are not independent. As expected, gene expression level and protein abundance are moderately correlated (Spearman's rho = 0.3, *p*-value<2e-16). Additionally, we noticed that protein length and abundance are inversely correlated to each other, with shorter proteins being more abundant (rho = −0.41, *p*-value<2e-16). Since we found that sampled proteins were longer but at the same time more abundant, correcting for protein length (by choosing a random subset of non-sampled proteins with the same length distribution as the set of sampled proteins) enhanced the difference in protein abundance even (Fig. S5). In addition, proteins that were sampled in both elution studies (*n* = 160) were found to be more abundant than source proteins that emerged only in one of the data sets (median abundance = 17.54 ppm (parts per million, see Materials and Methods) compared to 3.15 ppm, *p* = 1e-10). Moreover, we found a significantly higher rate of predicted binders (in the following referred to as the “predicted hit rate”) in sampled proteins, most pronounced for A*02:01-specific source proteins (median hit rate = 0.3 for sampled proteins vs. 0.025 for non-sampled proteins, *p* = 7e-14). Interestingly, within the same cell line, proteins that were sampled only by B*15:01 show a significantly lower predicted hit rate for A*02:01 than proteins that have been sampled by A*02:01 (median hit rate = 0.030 vs. 0.026, *p* = 7e-13), further emphasizing that the relative number of potential binding peptides does have an influence on sampling probability.

We do not have abundance data for vaccinia proteins available, but Assarsson *et al.* [24] measured vaccinia gene expression at several time points after infection. For each time point, we found a positive correlation between the gene expression level and the sampling frequency of the proteins when comparing single-sampled and multiple-sampled source proteins with those that did not give rise to eluted peptides (Fig. S6). Additionally, as for human proteins, sampled vaccinia proteins are longer than the remaining vaccinia proteins (*p*<0.01, data not shown).

### Putting it all together - prediction of protein sampling

**Figure 4:**
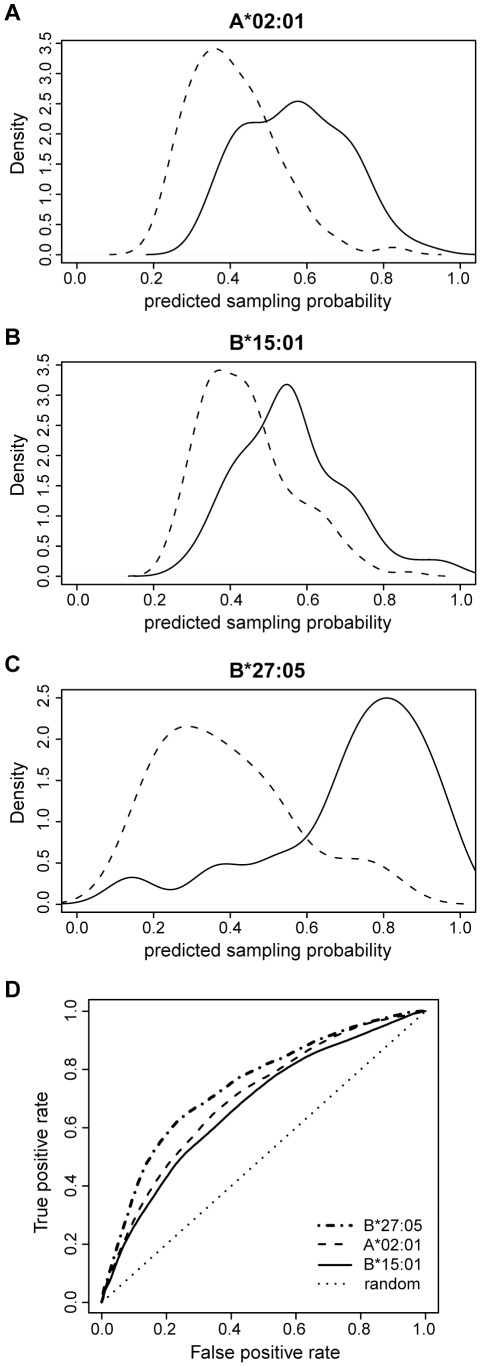
The regression model is able to distinguish sampled from non-sampled proteins. (**A**–**C**) Predicted sampling probability for A*02:01, B*15:01, and B*27:05 (best examples of 100 cross-validation runs per allele; solid line: sampled proteins; dashed line: non-sampled proteins). The sampling probability is calculated as f(*z*) = e*^z^*/(e*^z^*+1) where *z* = *c*+*c*_ab_ log10(*ab*)+*c*_pl_ *pl*+*c*_hr_ *hr* with *ab* the protein abundance, *pl* the protein length, *hr* the predicted hit rate, and (**A**) *c* = −1.47, *c*_ab_ = 0.49, *c*_pl_ = 0.0009, *c*_hr_ = 17.7, p-value = 5e-15, (**B**) *c* = −1.42, *c*_ab_ = 0.46, *c*_pl_ = 0.001, *c*_hr_ = 16.5, p-value = 1e-14, and (**C**) *c* = −1.77, *c*_ab_ = 1.15, *c*_pl_ = 0.0005, *c*_hr_ = 47.4, p-value = 1e-10. (**D**) Receiver operating characteristic (ROC) curve for A*02:01 (dashed), B*15:01 (solid), and B*27:05 (dash-dot) visualizing the performance of each of the regression models as a mean over 100 runs. The dotted line represents the ROC curve for random classification. Corresponding area under the curve (AUC): 0.70 for A*02:01, 0.68 for B*15:01, and 0.74 for B*27:05.

**Figure 5:**
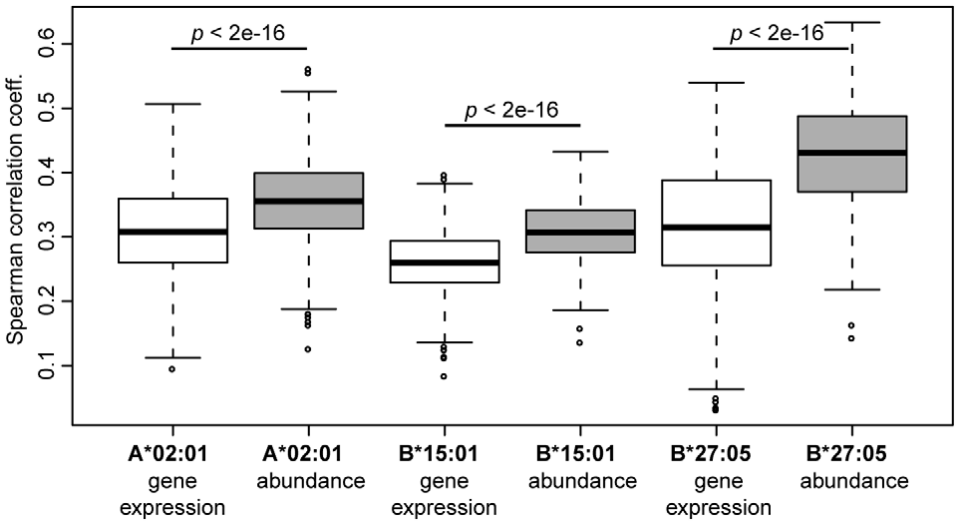
Protein abundance carries more information for the prediction of sampling probability than gene expression level. Boxplots of the Spearman correlation coefficients resulting from one hundred 5× cross-validation runs for regression models that either include gene expression data or protein abundance data.

Overall, all tested factors - protein length, gene expression level, protein abundance, and predicted hit rate - show differences between the set of sampled proteins and the proteins that were not sampled. In order to quantify the contribution of each factor and to determine which combination of factors best describes the data, we performed a multiple logistic regression analysis. Starting from a maximal model including all factors as explanatory variables, we obtained a minimal model by iterative exclusion of non-significant factors. Before running the regression, we first randomly picked a subset of non-sampled proteins to form a “negative” set of equal size as the positive set of sampled proteins. This balanced set of negative and positive data points was then used to perform a logistic regression and performance analysis (see Materials and Methods), which was repeated 100 times with different random negative subsets. The performance was measured as the Spearman correlation coefficient between the known sampling status (i.e., a binary value) and the predicted sampling probability. For all three HLA allotypes, a regression model combining protein abundance, protein length and predicted hit rate showed the best performance (the best examples are given in Fig. 4A–C). Since we found that eluted peptides are more efficiently processed than other HLA-binding peptides, we tested whether we could improve the model by filtering the set of predicted binders for processing efficiency. For all three HLA allotypes, this filtering step improved the prediction performance only to a minor extent (results not shown). As gene expression and protein abundance are moderately correlated, we tested which of these two factors would carry more information for predicting protein sampling. We found that protein abundance clearly outcompetes gene expression (Fig. 5). Among the three HLA allotypes, the prediction performance of the B*27:05 model was best (Fig. 4C), with an average AUC (area under the receiver operating characteristic curve [25]) value of 0.74 compared to 0.70 for A*02:01 and 0.68 for B*15:01 (Fig. 4D). Overall, the resulting logistic regression models were able to discriminate between sampled and non-sampled proteins to a significant degree (Fig. 4A–C).

## Discussion

Only a small fraction of the human proteome is sampled by the class I antigen processing pathway and presented on HLA class I molecules. Previously it was suggested that the cellular localization of a protein and its function play a role in this sampling process [8], [23]. Here we show that other protein characteristics like protein length, abundance, and rate of predicted binders also largely influence the sampling probability of a protein and thereby shape the peptide repertoire of an HLA class I molecule.

We analyzed two large peptide elution datasets; one derived from a vaccinia virus infected cell line, and one obtained from cells transfected with a gene encoding a soluble HLA class I molecule. Identification of the source protein of each peptide showed that, in spite of the huge difference in proteome size between human and vaccinia virus, a similar fraction of either proteome (10–12% of all proteins) was sampled by the antigen processing pathway. We characterized the set of eluted peptides in terms of antigen processing and presentation and observed a significantly higher binding affinity to the respective HLA molecule and more efficient processing for the eluted peptides than for other potential binders derived from the same set of source proteins. The predicted median affinity of eluted peptides was 14 nM IC50 for A*02:01, 65 nM IC50 for B*15:01, and 107 nM IC50 for B*27:05. These values are much lower than the 500 nM, which are often used as a threshold to discriminate HLA-binding peptides from non-binders [26]. This observation could reflect that high-affinity binders are preferentially loaded onto the HLA molecule among others with the help of the ER resident chaperone tapasin [27], [28], or that they have a longer “life span” on the cell surface because they form a more stable complex with the HLA molecule, which increases their chance of being eluted (even though this is rather related to the off-rate of a peptide than to its affinity). Especially for the elution studies involving soluble HLA molecules, it is not surprising to identify foremost high-affinity binders after the long affinity purification process [14]. A higher binding affinity of eluted peptides has also been found for mouse MHC class I molecules [12]. If high-affinity peptides are able to outcompete lower affinity-peptides in binding to the HLA, this may result in a higher copy number of these peptides on the cell surface which in turn increases their chance to be detected by mass spectrometry. The nature of the data sets we analyzed does not allow us to study this because we do not have abundance data on the peptides. Instead we merely know whether a peptide was present in the eluate or not.

To our surprise, most proteins were represented by only a single peptide in the elution data sets we studied. This is in line with the observation by Hickman *et al.* [8] who found only 9 of 189 source proteins (4.8%) to be represented by more than one peptide.

For the prediction of C-terminal processing, we employed a method that has been trained on the C-termini of known HLA ligands [17]. Initially intended as a predictor of proteasomal cleavage, the method automatically accounts for the contribution of other potential peptidases that are able to further process the carboxy terminus of proteasome products, as for example the carboxypeptidase ACE [6]. It does, however, not account for the activity of aminopeptidases in the cytosol and ER, which may further trim the amino termini of peptides. There is some evidence for the existence of N-terminal processing motives, which differ in specificity between cytosol and ER [29], [30]. However, the lack of appropriate prediction methods prevented us from assessing the effect of N-terminal trimming of peptides in our analysis.

For the data sets we studied, we observed that (i) source proteins are longer and more abundant than non-source proteins, (ii) the corresponding genes show higher expression levels, and (iii) source proteins show a higher rate of predicted binders than proteins that were not sampled. We combined these factors in a logistic regression model and conclude that prediction of protein sampling probability is possible to some degree. The best model made use of protein length, abundance, and predicted hit rate to predict the sampling probability of a protein. Fortier *et al.* [12] observed that MHC-presented peptides are preferentially derived from highly abundant mRNAs. Our analysis confirmed the impact of gene expression reported earlier by Fortier *et al.*, but in addition, our results suggest that protein abundance carries more information for the prediction of protein sampling than transcript levels do.

It has been argued that antigen processing should be correlated with protein turnover rather than cellular abundance of proteins [10]. In addition, a recent study suggested that the pioneer round of mRNA translation, which serves as a “proof-reading” step during mRNA maturation, might be a major source of HLA ligands [31]. We believe that the model presented in this paper will improve considerably when more data is available describing the specificity and kinetics of peptide generation via these processes. Finally, another source of antigenic peptides are so-called defective ribosomal products (DRiPs), which are truncated and/or misfolded polypeptides that are directly targeted to proteasomal degradation [32], [33]. The DRiP hypothesis suggests that the set of MHC-presented peptides reflects recent protein synthesis rather than the protein content of the cell, which should manifest itself in our analysis as a higher correlation with gene expression level than with protein abundance. Even though this is not what we see, the fact that HLA ligands are preferentially derived from long proteins is in accordance with the DRiP hypothesis, because the chance of incorporating errors and of misfolding increases with protein length.

A limitation of our analysis is the presumably high noise in the protein abundance and gene expression data. The abundance data was derived through meta-analysis from a multitude of different tissue types, even though there is considerable variation of protein abundance between cells and tissues. However, as Weiss *et al.* [34] report, the abundance data set consists mainly of house-keeping genes whose tissue-to-tissue expression variability is limited. Ideally, the analysis presented here should be repeated on a data set where mRNA levels, protein abundance, and HLA peptide presentation are measured simultaneously for a single cell type or tissue sample to minimize noise. All the more striking it is, however, that we see a clear signal for both gene expression and protein abundance in their impact on protein sampling in spite of the noise introduced by averaging over cell types.

In conclusion, the results presented in this paper demonstrate that protein characteristics such as gene expression level, protein abundance, and the rate of HLA ligands determine which protein will be sampled for antigen presentation. Moreover, our results suggest that sampling prediction may be extended to the proteomes of pathogens, allowing us to identify promising targets for vaccination studies.

## Materials and Methods

### Elution data sets

Johnson *et al.* [13] performed peptide elution and mass-spectrometry analysis of vaccinia virus infected Epstein-Barr virus-transformed B-cells, homozygous for HLA-A*02:01, B*15:01, and C*03 (for details see [13]). With a false positive rate (FPR) of 5%, they identified 4717 unique human-derived peptides and 119 vaccinia derived peptides.

Ben Dror *et al.* [14] eluted peptides from cultured cartilage cells and HeLa cells transfected with a soluble form of HLA-B*27:05. Based on several criteria to assess the confidence in identified peptides, they categorized eluted peptides into three subsets: certain (569 peptides), probable (582 peptides), and possible (116 peptides). As the *certain* peptide set corresponds to a FPR of 4.7%, we limited our analysis to this data set. Of note, in the original publication, peptides were selected as correct only if they contained the amino acids arginine or glutamine at their second position [14], which according to the authors (personal communication) was necessary in order to filter out peptides that were eluted from other HLA allotypes expressed by the cell line (which may become soluble due to cellular stress).

### Identification of source proteins

We obtained the human proteome from Ensembl Genomes (ftp.ensembl.org/pub/, release 56) and used this collection of proteins to identify the source protein for each peptide in our elution data sets. Source protein identification required identical mapping of a peptide to the source protein sequence. Peptides that could not be uniquely mapped to one single protein were omitted from further analysis. In the case of several splice variants of the same protein (i.e., the peptide matched to several protein sequences which all originate from the same gene), the longest splice variant was chosen for sequence analysis. Likewise, the longest splice variant was chosen for the set of non-sampled proteins. We were able to map 105 of the vaccinia peptides to the Vaccinia Western Reserve proteome (GenBank identifier: AY243312).

### Abundance and gene expression data

We had abundance data available for 12,021 human proteins [34]. The abundance is expressed in parts per million (ppm), relative to the molecule counts of all other proteins in the proteome. The measured abundance for different proteins spans several orders of magnitude. The protein abundance data covers 1986 (78.4%) of the 2533 *Johnson* source proteins and 340 (85.8%) of the 396 *Ben Dror* source proteins.

We used gene expression data from Juncker *et al.* [23], who provide the median of normalized mRNA levels of haematopoietic tissues originally obtained from the GNF gene expression database [35]. Expression levels of vaccinia virus genes were obtained from Assarsson *et al.* [24].

### Prediction methods

Throughout the study we used the method NetMHC 3.2 [15], [16] to predict peptide-binding to the HLA molecules A*02:01, B*15:01, and B*27:05. Binding predictions for C*03 were done using NetMHCpan 2.4 [36]. We define predicted binders as peptides that have a predicted binding affinity of <500 nM IC50 for a particular HLA molecule. For the *Ben Dror* data, where all identified peptides were eluted from a known HLA molecule (namely, HLA-B*27:05), this proved to be a suitable threshold, predicting 89.6% (510 of 569) of the eluted peptides as binders.

The NetChop version Cterm-3.0 [17], [37] was used for the prediction of C-terminal processing. Furthermore, we used weight matrices provided by the mhc-pathway package [19], [20] for the prediction of cleavage probability by the immunoproteasome and for TAP transport efficiency. We employed WoLF PSORT [38] to predict subcellular localization of proteins and confirmed our results by GO-term enrichment analysis using the Cytoscape [39] plug-in Bingo [40].

### Predicted hit rate

The predicted hit rate for a given protein is defined as the ratio of the number of predicted binders for a particular HLA allotype to the total number of unique 9mer peptides in this protein. Multiple occurrences of the same peptide within one protein were counted as a single occurrence, because they would also not be detected as separate peptides in the elution analysis. The hit rate is calculated per HLA allele and hit rates may differ between alleles, because we use a fixed affinity threshold of 500 nM IC50 to define binders (instead of assigning a fixed fraction of peptides, e.g. top 1%, as binders). For this reason we did not directly compare hit rates between alleles, but instead performed separate analyses per HLA allele.

### Statistical analysis

Two-sided Mann-Whitney tests, correlation tests, Chi-squared tests, and logistic regression analysis were performed using R (http://www.R-project.org). We used a generalized linear model with a binomial response distribution and a logit function for data transformation to model the impact of various factors on sampling probability. All figures were produced using R.

## Supporting Information

Figure S1 Eluted peptides show a significantly higher C-terminal processing probability than other predicted binders. (**A**) In order to normalize the peptide data sets for predicted binding affinity, for each HLA allotype, we picked an affinity-matched subset of predicted binders so that the range of predicted binding affinities was the same as the range for eluted peptides. (**B**) After normalizing for the binding affinity, eluted peptides still show a significantly higher C-terminal processing probability.
(TIF)

Figure S2 Eluted peptides are more likely to be produced by the immunoproteasome and are more efficiently transported by TAP. The boxplots compare eluted 9mer peptides and predicted binders from the same set of source proteins in terms of (**A**) predicted C-terminal cleavage probability by the immunoproteasome and (**B**) predicted TAP transport efficiency. Here, the eluted peptides are compared to all predicted binders originating from the same set of source proteins. Similar results are obtained when using an affinity-matched subset of predicted binders (cf. Fig. S1).
(TIF)

Figure S3 Eluted vaccinia peptides show a significantly higher (**A**) predicted binding affinity to A*02:01 and B*15:01, respectively, and (**B**) predicted C-terminal processing probability (for A*02:01-eluted peptides) than other predicted binders from the same set of vaccinia proteins.
(TIF)

Figure S4 Distribution of predicted cellular compartments for all human proteins and the source proteins identified for the *Johnson* data and the *Ben Dror* data. Subcellular localization as given by WoLF PSORT (nucl = nucleus, mito = mitochondria, extr = extracellular, ER = endoplasmic reticulum, cyto_nucl = cytosol and nucleus, cyto = cytosol, plas = plasma membrane). Proteins targeted to the extracellular compartment were underrepresented with 6% for the *Johnson* and 8% for the *Ben Dror* data compared to 20% among all human proteins (*p*<2e-16 and *p* = 5e-10, respectively, Chi-squared test), whereas cytosolic proteins were overrepresented among the sampled proteins (27%–28% vs. 18%, *p*<2e-05). These results were confirmed by a GO-term enrichment analysis performed using the Cytoscape plug-in Bingo, which identified a significant underrepresentation of GO-terms relating to the plasma membrane (19% among sampled vs. 32% among all human proteins) and the extracellular compartment (4.5% vs. 12.5%), while revealing an enrichment of intracellular proteins (93% vs. 70%).
(TIF)

Figure S5 Comparison of sampled and non-sampled human proteins in terms of protein abundance after normalization for protein length. Normalization was achieved by choosing a random subset of non-sampled proteins that show the same length distribution as the set of sampled proteins.
(TIF)

Figure S6 The gene expression level of vaccinia genes is correlated with the sampling state (none, one, or several peptides found by elution). The gene expression level was measured at indicated time points after infection by Assarsson *et al.* (2008).
(TIF)
