# Supplementary figures and images for: Proteome Sampling by the HLA Class I Antigen Processing Pathway
Source: PLoS Comput Biol. 2012 May 17;8(5):e1002517. doi: 10.1371/journal.pcbi.1002517 (PMC3355062; doi:10.1371/journal.pcbi.1002517)

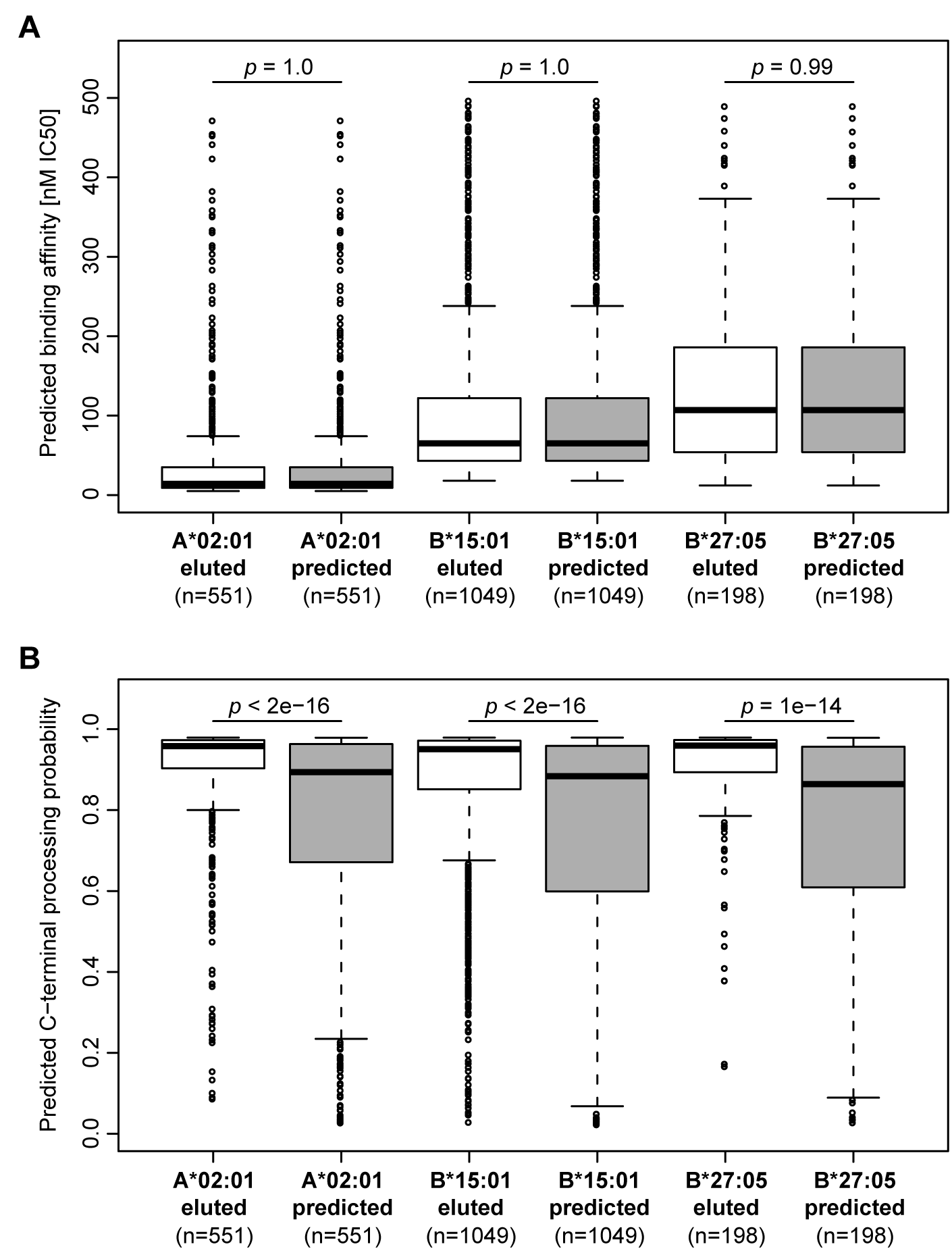

Supplement: Figure S1 — Eluted peptides show a significantly higher C-terminal processing probability than other predicted binders. (A) In order to normalize the peptide data sets for predicted binding affinity, for each HLA allotype, we picked an affinity-matched subset of predicted binders so that the range of predicted binding affinities was the same as the range for eluted peptides. (B) After normalizing for the binding affinity, eluted peptides still show a significantly higher C-terminal processing probability. (TIF) [file pcbi.1002517.s001.tif]

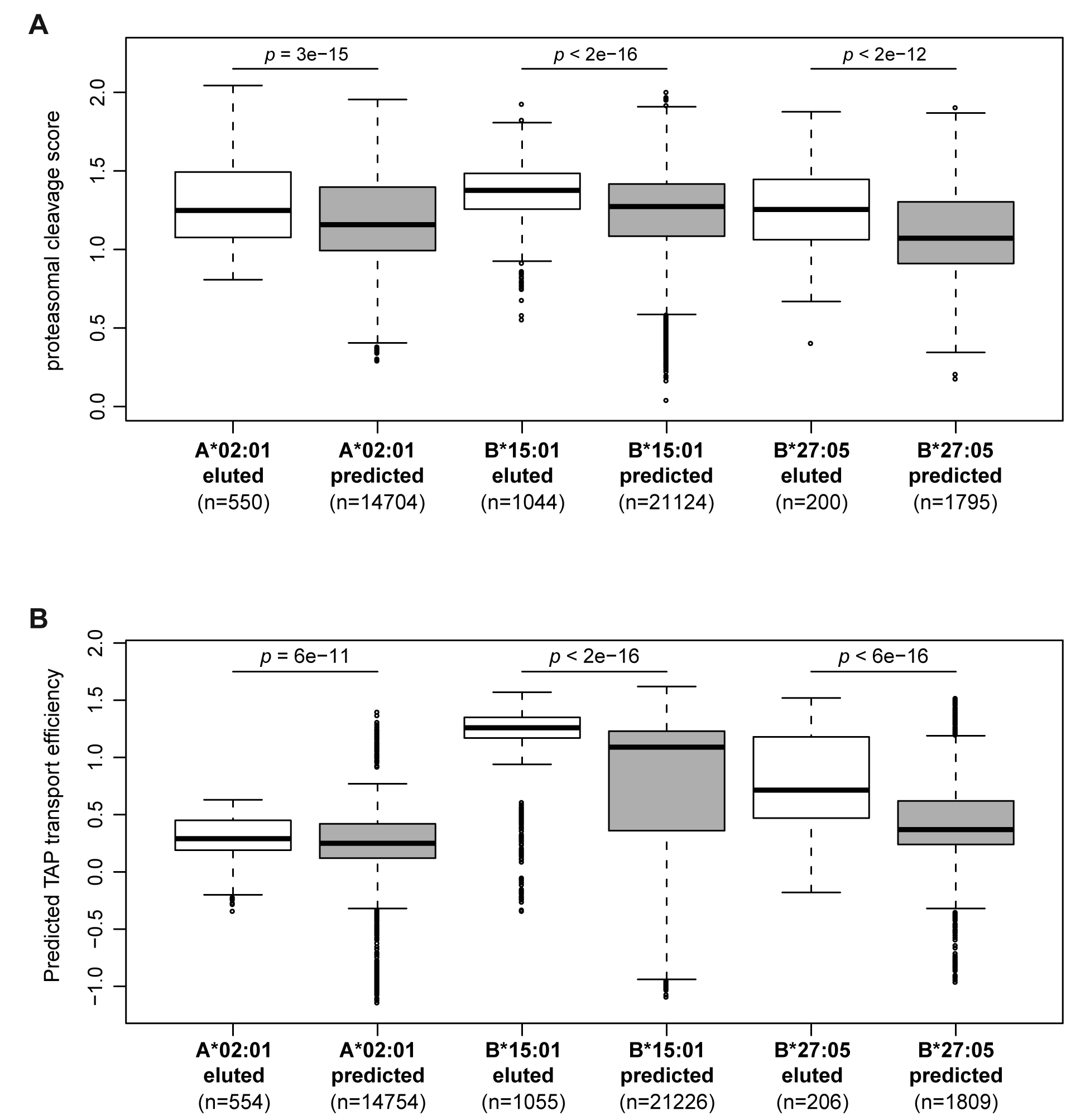

Supplement: Figure S2 — Eluted peptides are more likely to be produced by the immunoproteasome and are more efficiently transported by TAP. The boxplots compare eluted 9mer peptides and predicted binders from the same set of source proteins in terms of (A) predicted C-terminal cleavage probability by the immunoproteasome and (B) predicted TAP transport efficiency. Here, the eluted peptides are compared to all predicted binders originating from the same set of source proteins. Similar results are obtained when using an affinity-matched subset of predicted binders (cf. Fig. S1). (TIF) [file pcbi.1002517.s002.tif]

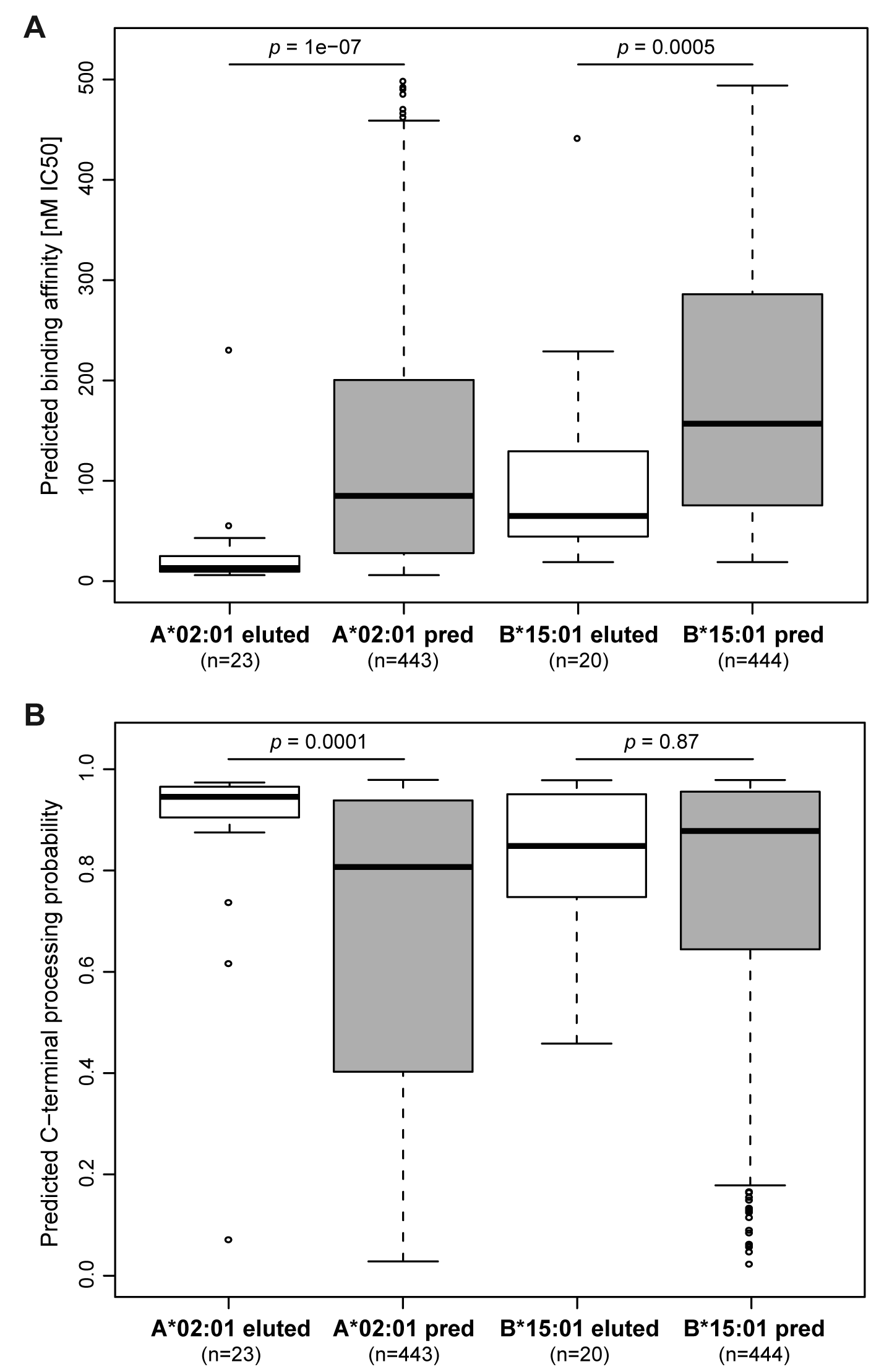

Supplement: Figure S3 — Eluted vaccinia peptides show a significantly higher (A) predicted binding affinity to A*02:01 and B*15:01, respectively, and (B) predicted C-terminal processing probability (for A*02:01-eluted peptides) than other predicted binders from the same set of vaccinia proteins. (TIF) [file pcbi.1002517.s003.tif]

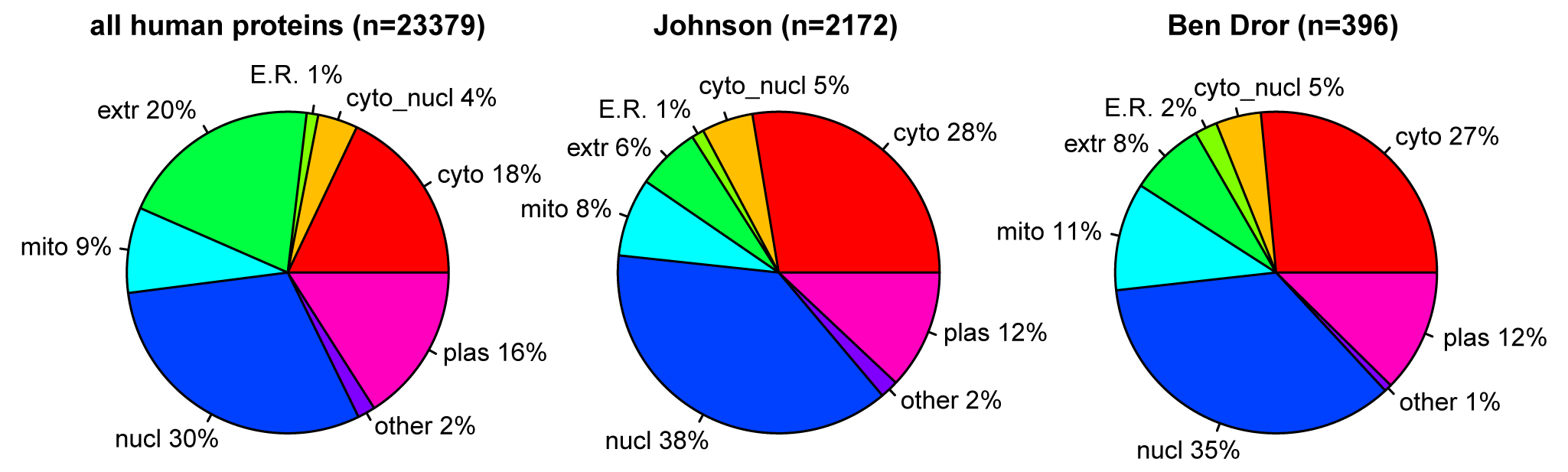

Supplement: Figure S4 — Distribution of predicted cellular compartments for all human proteins and the source proteins identified for the Johnson data and the Ben Dror data. Subcellular localization as given by WoLF PSORT (nucl = nucleus, mito = mitochondria, extr = extracellular, ER = endoplasmic reticulum, cyto_nucl = cytosol and nucleus, cyto = cytosol, plas = plasma membrane). Proteins targeted to the extracellular compartment were underrepresented with 6% for the Johnson and 8% for the Ben Dror data compared to 20% among all human proteins (p<2e-16 and p = 5e-10, respectively, Chi-squared test), whereas cytosolic proteins were overrepresented among the sampled proteins (27%–28% vs. 18%, p<2e-05). These results were confirmed by a GO-term enrichment analysis performed using the Cytoscape plug-in Bingo, which identified a significant underrepresentation of GO-terms relating to the plasma membrane (19% among sampled vs. 32% among all human proteins) and the extracellular compartment (4.5% vs. 12.5%), while revealing an enrichment of intracellular proteins (93% vs. 70%). (TIF) [file pcbi.1002517.s004.tif]

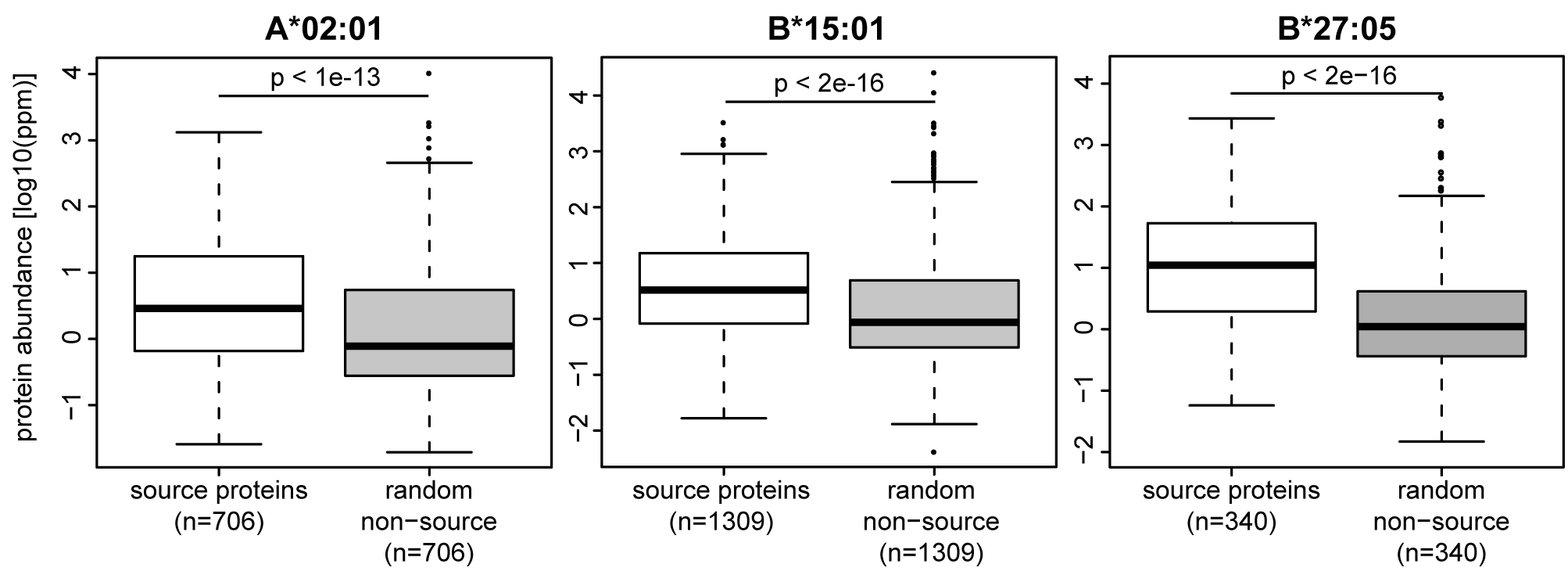

Supplement: Figure S5 — Comparison of sampled and non-sampled human proteins in terms of protein abundance after normalization for protein length. Normalization was achieved by choosing a random subset of non-sampled proteins that show the same length distribution as the set of sampled proteins. (TIF) [file pcbi.1002517.s005.tif]

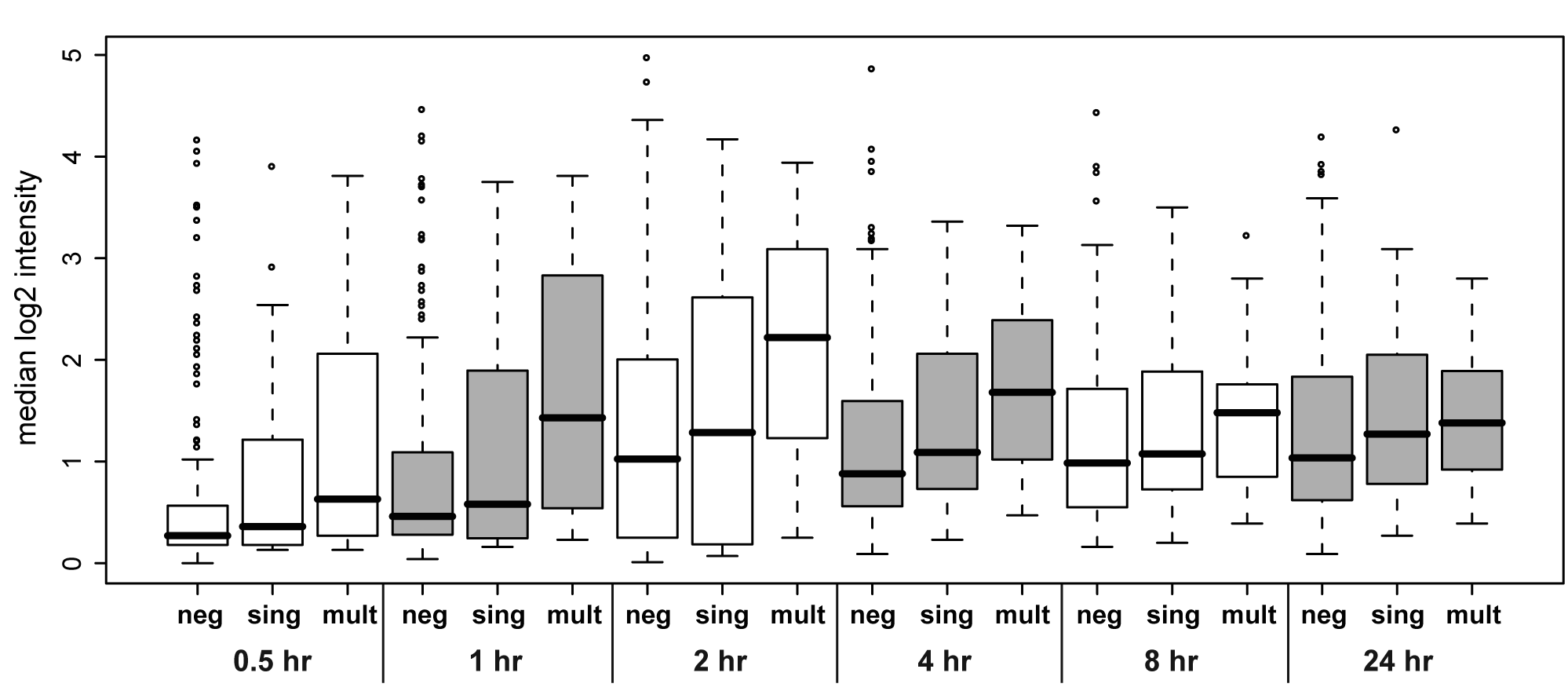

Supplement: Figure S6 — The gene expression level of vaccinia genes is correlated with the sampling state (none, one, or several peptides found by elution). The gene expression level was measured at indicated time points after infection by Assarsson et al. (2008). (TIF) [file pcbi.1002517.s006.tif]
